# Effects of continuous cropping on fungal community diversity and soil metabolites in soybean roots

**DOI:** 10.1128/spectrum.01786-23

**Published:** 2023-10-09

**Authors:** Dexin He, Xingdong Yao, Pengyu Zhang, Wenbo Liu, Junxia Huang, Hexiang Sun, Nan Wang, Xuejing Zhang, Haiying Wang, Huijun Zhang, Xue Ao, Futi Xie

**Affiliations:** 1 Soybean Research Institute, Shenyang Agricultural University, Shenyang, China; 2 Postdoctoral Station of Agricultural Resources and Environment, Land and Environment College, Shenyang Agricultural University, Shenyang, China; 3 Inner Mongolia Agronomy and Animal Husbandry Technology Extension Center, Hohhot, Inner Mongolia, China; Pátzcuaro, Michoacán, Mexico

**Keywords:** soybean, continuous cropping, pathogenic fungus, soil metabolites

## Abstract

Microbial community imbalance is the main cause of soybean intercropping, but the mechanism of soybean soil fungal community diversity change induced by continuous cropping is still unclear. This study analyzes the fungal community diversity and the change of fungal communities in compartments of different root systems of intercropped soybeans by high-throughput sequencing (endosphere, rhizoplane, and rhizosphere) between continuous cropping and maize-soybean rotation. The results showed that the community composition and the diversity of compartments of different root systems of intercropped soybeans are different, and fungal diversities showed a decreasing trend from rhizosphere to endosphere. Continuous cropping significantly increased fungal community diversities in different root compartments and changed their formation, enrichment, and depletion processes. Continuous cropping brings about the enrichment of soil pathogens, but only partial soil pathogens could colonize from the rhizosphere to the endosphere. All these suggested that root compartments had selective effects on root-associated fungal community diversity. Additionally, metabolomics assay revealed that continuous cropping soybean markedly altered soil metabolic profiling. Correlation analysis results showed that fungal community diversity was significantly correlated with soil metabolites. To sum up, under different planting patterns, the diversity and the functional mode of soybean soil microbial community have changed. Continuous cropping has increased the abundance of pathogenic fungi in soybean soil, resulting in changes in the correlation between soil fungi and metabolites, and then changed the soil metabolic spectrum.

**IMPORTANCE:**

Soybean yield can be affected by soybean soil fungal communities in different tillage patterns. Soybean is an important food crop with great significance worldwide. Continuous cultivation resulted in soil nutrient deficiencies, disordered metabolism of root exudates, fungal pathogen accumulation, and an altered microbial community, which brought a drop in soybean output. In this study, taking the soybean agroecosystem in northeast China, we revealed the microbial ecology and soil metabolites spectrum, especially the diversity and composition of soil fungi and the correlation of pathogenic fungi, and discussed the mechanisms and the measures of alleviating the obstacles.

## INTRODUCTION

Approximately 42% of China’s total acreage is devoted to the soybean crop area in the Northeast (1). Due to arable land area, short crop growing season, market demand, and intensive cultivation of modern agriculture, continuous cropping soybean is common in this area (2, 3). Continuous cultivation resulted in soil nutrient deficiencies (4), disordered metabolism of root exudates (5), fungal pathogen accumulation (6), and altered microbial community (3, 7, 8), which brought a drop in soybean output. Juo (9) found that compared with unsterilized soils grown continuously, soil sterilization significantly improved the growth of soybean plants, indicating that biological factors were an important incentive for continuous soybean barriers (10). Later, researchers gave more attention to study microorganisms in continuous cropping soybean. Wang and Liu (11, 12) discovered that continuous cropping brought about a rise in the wealth of fungal pathogens, and a shift in fungal community structure, leading to decreased soybean yield.

Soil fungi were divided into harmful and beneficial fungi on the basis of their functions within the agroecosystem (12). Enrichment of pathogenic fungi in crops causes diseases in crops, while enrichment of beneficial fungi in crops inhibits fungal pathogens and enhances the growth of crops (13). Continuous cropping brought about the enrichment of phytotoxic root exudates (5), which altered the formation of the microbial community and brought about enrichment in the abundance of harmful fungi (11). A study by Xiong (14) confirmed that continuous cropping altered fungal variety and obviously added plenty of *Thanatephorus*, *Fusarium,* and *Alternaria*, which caused pathogen emergence. Continuous cropping also resulted in the decline of beneficial fungi. Continuous cropping soybean reduced the quantities of *Trichoderma* and *Gliocladium*, which played roles in the preventive treatment of harmful soil fungi (15). It is therefore of great importance to agricultural development to strengthen research into the soil fungal community of continuous cropping soybean.

In order to investigate plant-microbe interactions, Edwards (16) further divided the root system into three compartments through different spatial scales, both horizontally and vertically (endosphere, rhizoplane, and rhizosphere); the results have shown that microorganisms from different ecological niches are different and play different roles within the soil ecosystem. Plant roots recruit specific microorganisms to the rhizosphere from the bulk soil, and microorganisms and plants have complex interactions that are directly involved in plant soil reproductive growth, health, and other aspects (17). Microorganisms that colonized the rhizosphere of plants provided a number of beneficial functions to their host (18). Besides, they confirmed that community diversity of the fungal along the soil-plant continuum is very important for the plant-microbial interaction relationship (19). Root niches exerted selective effects on the fungal community diversity of the root-associated microbiome (16, 20), but our understanding of such mechanisms of selection under continuous cropping soybean remained largely unknown.

By using soil metabolomics techniques, changes in soil internal structure can be observed (21), and the joint research of metabolomics and microbiomics can better understand the changes in the internal environment of soil (22). There was a very close relationship between soil microorganisms and soil metabolites. Soil metabolites can provide important substances for soil microorganisms' life activities, and soil microorganisms can further come into contact with plant bodies to produce secondary metabolites (23). In the rhizosphere, the increase in acids, organic compounds, lipids, and other substances can easily increase the number of harmful microorganisms in the soil (24). However, the mechanism behind the interaction between soybean fungi and soil metabolites in continuous cropping has not yet been studied and understood.

The objectives of this study were to combine ITS sequencing and untargeted metabolomics to (i) investigate the response of rhizosphere fungal assemblage and soybean soil metabolites to continuous cropping and (ii) to clear the relationships and theories of soil fungal and metabolite interactions in continuous cropping using a combination of soil microbiome and metabolomics analysis.

## MATERIALS AND METHODS

### Site and sampling

In 2021, the experiment was conducted in the Liaozhong location (122.73°E, 41.51°N), Liaoning province, China. Two treatments were implemented: continuous cropping soybean and corn-soybean rotation. Liaodou 14 was used as the experimental cultivar. Each plot consisted of 5 rows, 18 m in length, 0.6 m apart from the ridge, 150,000 plants/ha in density, 3 replicates, and 2 plants per hole, with the remainder being conventional field management.

Samples were selected using the five-point method. In brief, five plots (5 cm × 20 cm) were chosen at random from each treatment. Sift 2 mm of soil to remove plant debris, leftover tissue, gravel, etc., and divide it into three equal parts. During the soybean grain filling stage (R6), a sterile brush was exercised to take soil within 5 mm of the surface of the soybean root as rhizosphere soil. Plant roots harvested from the rhizosphere were cut and placed in sterile water at a strength of 100 r/min for 30 min. Soils dissolved in water were used as rhizoplane. Root surfaces were removed from root systems as samples of endophytic bacteria. The samples were mixed and divided into three parts for sequencing analysis, while the remainder, ultra-cold, were stored for subsequent assays.

### Soil fungal community diversity

#### Methods: sequencing preparation

Total DNA was extracted from 0.7 g of soil samples and 0.5 g of roots using BioFast Soil Genomic DNA Extraction kit (BioFlux, Hangzhou, China) according to the manufacturer’s instructions. DNA concentration was detected using Nanodrop 2000C Spectrophotometer (Thermo Fisher Scientific, United States). For the fungal community, the primer set ITS5-1737F (5′-GGAAGTAAAAGTCGTAACAAGG-3′)/ITS2-2043R (5′-GCTGCGTTCTTCATCGATGC-3′) were chosen to target the fungal ITS1 region. The PCRs were carried out by Novogene Co., Ltd. (Beijing, China) using Phusion R High-Fidelity PCR Master Mix with GC Buffer, 0.2 µM of forward and reverse primers, and about 10 ng template DNA. Thermal cycling consisted of initial denaturation at 98℃ for 1 min, followed by 30 cycles of denaturation at 98°C for 10 s, annealing at 50℃ for 30 s, elongation at 72°C for 30 s, and, finally, elongation at 72°C for 5 min. PCR products were mixed in equidensity ratios. Then, the mixture of PCR products was purified with Qiagen Gel Extraction Kit (Qiagen, Germany). The library was then sequenced on an Illumina HiSeq2500 platform.

#### OTU clustering and species annotation

Each sample gets Tags data, through (https://github.com/torognes/vsearch/) (25), and the species annotation database compares to get the Effective Tags. The Uparse algorithm (Uparse v7.0.1001, http://www.drive5.com/uparse/) (26) was used for Effective Tags clustering of all samples. By default, sequences are clustered into Operational Taxonomic Units (OTUs) with 97% consistency. OTU results and research requirements were obtained according to clustering, common and unique OTUs among different samples (groups) were analyzed, and the petal map was drawn. Qiime software (Version 1.9.1) blast method (http://qiime.org/scripts/assign_taxonomy.html) (27) and Unit (v8.2) database (https://unite.ut.ee/) (28) were used for species annotation analysis, and at each taxonomic level, kingdom, phylum, class, order, family, genus, and species were counted for the community composition of each sample. A functional taxonomic heat map was developed based on species annotations and abundance information for all samples at the genus level. Based on the species abundance, according to the filtered value of phase relationship, with bacteria as node and value as edge, graphviz-2.38.0 was used to draw the network diagram.

### Soil metabolite analyses

After vacuum freeze-drying, the collected rhizosphere soil was ground to powder, and 0.5 g sample was weighed and added to 1 mL methanol, isopropyl alcohol, and water extract with a volume ratio of (3:3:2). The samples were shaken at room temperature for 3 min, followed by ultrasound in an ice water bath for 20 min. At 4°C, the sample was centrifuged at 12,000 r/min for 3 min, the supernatant was collected in the sample bottle, 0.02 mL of internal standard solution (10 µg/mL) was added into the bottle, the sample was blown dry with a nitrogen blower, and it was placed in the freeze-drying machine. Add 0.1 mL of methoxamine salt pyridines with a concentration of 0.015 g/mL and oxime at 37°C for 2 h, then add 0.1 mL of BSTFA (with 1% TMCS), and put in a 37°C oven for the reaction for 0.5 h to obtain a derivative solution for GC-MS detection.

PCA normalized the data with R software for unit variance scaling (UV). R software ComplexHeatmap package is used to draw heat maps, and hierarchical clustering analysis is conducted on the accumulation patterns of metabolites in different samples. The built-in cor function of R software was used to calculate Pearson’s correlation coefficient and analyze the correlation between samples. KEGG database (29) was used to annotate differential metabolites and explore the interaction of differential metabolism in organisms.

### Data processing

The data were processed by Excel 2010, the statistical software SPSS (IBM SPSS Statistics v24) was used for analyzing the significant difference, and Duncan’s new multiple range test was used.

## RESULTS

### Effect of continuous cropping on fungal community diversity and composition in different root compartments

**Fig 1:**
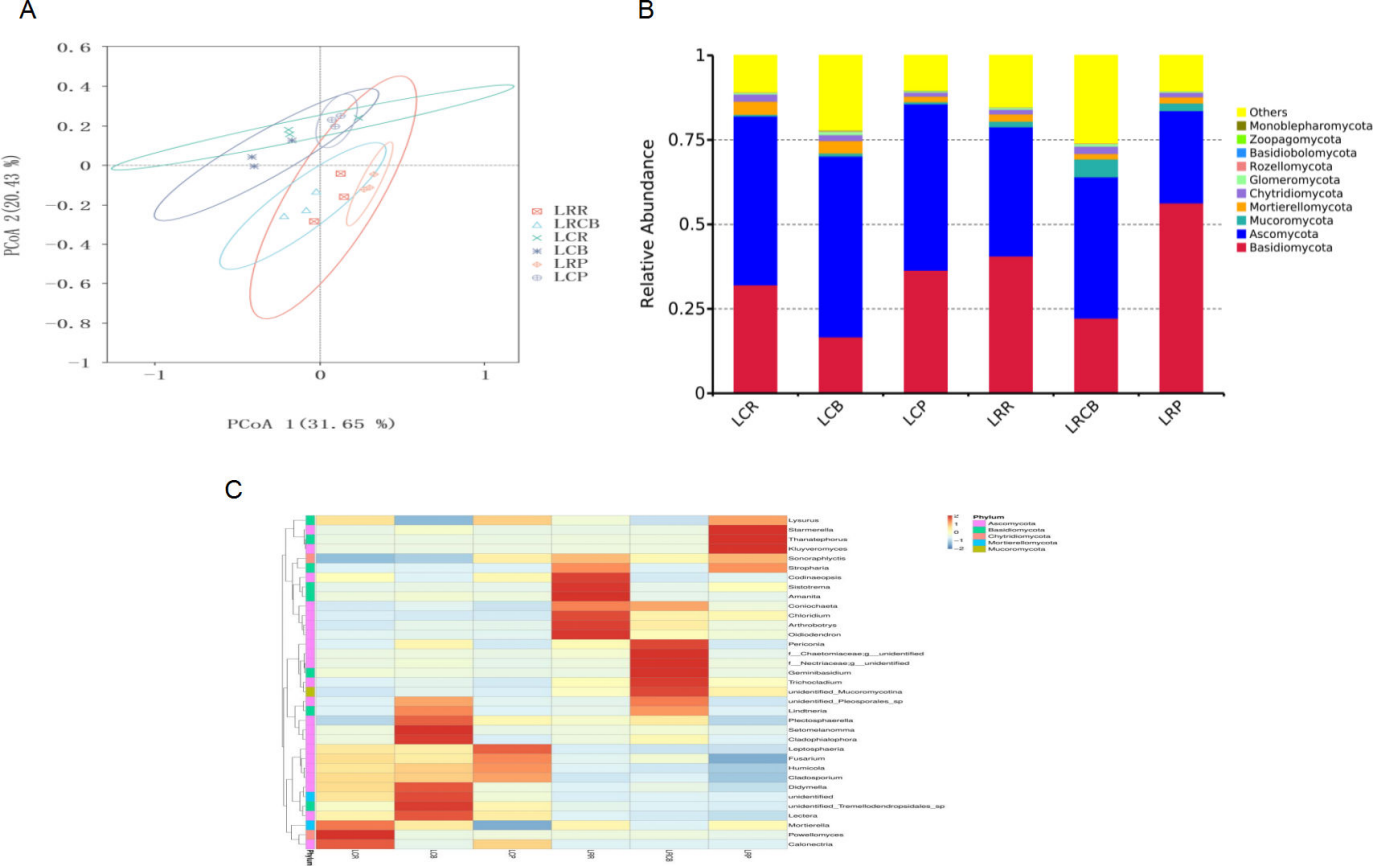
Diversity and composition of three fungal niches in the soybean root system in one rotation of continuous cropping soybean and maize-soybean. (A) Primary coordinate analysis (PCOA) of fungal communities was conducted and grouped according to three ecological sites related to the root. (B) Relative abundance of the top 10 species at the phyllographic level. (C) Cluster abundance heat map of the horizontal species of the genus. LCB, continuous cropping rhizosphere; LCR, continuous cropping rhizoplane; LCP, continuous cropping endosphere; LRCB, rotation rhizosphere; LRR, rotation rhizoplane; LRP, rotation endosphere. (*P* < 0.05).

Principal coordinate analysis (PCoA) showed that the fungal communities of continuous cropping soybean and rotation of corn and soybean were well separated (*P* < 0.05), and the fungal communities in the different root compartments were also well separated (*P* < 0.05) (Fig. 1A).

The top 10 dominant phyla in the fungal communities across all soil samples were *Basidiomycota, Ascomycota, Mucoromycota, Mortierellomycota, Chytridiomycota, Glomeromycota, Rozellomycota, Basidiobolomycota, Zoopagomycota, and Monoblepharomycota* (Fig. 1B). In continuous cropping soybean and maize-soybean rotation, the abundance of *Basidiomycota* increased gradually from the rhizosphere to the endosphere, whereas the abundance of *Ascomycota* decreased. Continuous cropping increased the abundance of *Basidiomycota* and decreased the abundance of *Ascomycota*, while rotation increased the abundance of *Ascomycota* and decreased the abundance of *Basidiomycota*. The abundance of *Mortierellomycota, Glomeromycota, Zoopagomycota,* and *Monoblepharomycota* was also increased by continuous cropping. The enrichment of *Ascomycota* and *Mortierellomycota* in LCR was the highest, while the enrichment of *Mucoromycota* and *Chytridiomycota* in LRCB was the highest.

At the genus level (Fig. 1C), the top 10 most dominant genera were *Lysurus*, *Stropharia*, *Fusarium*, *Calonectria*, *Kluyveromyces*, unidentified_*Mucoromycotina*, *Lindtneria*, *Plectosphaerella*, *Codinaeopsis*, and *Didymella*. The abundance of *Fusarium*, *Calonectria*, *Kluyveromyces*, *unidentified_Mucoromycotina, Lindtneria*, *Plectosphaerella*, *Codinaeopsis*, and *Didymella* was increased by continuous cropping. *Fusarium* was enriched at 20.70%, 18.99%, and 25.11% in the LCR, LCB, and LCP samples, respectively. Significant differences were observed between different root compartments. *Lysurus* was more enriched in LRP and LCP than the others, while the enrichment of *Stropharia* in the LRR and LRP was greater than the others.

### Enriched or depleted fungal OTUs in different root compartments

In order to identify OTUs that correlate with the fungal community in different compartments, differential abundance analyses were performed using a negative binomial distribution with counts of OTUs. Rhizosphere soil was set as a control and adjusted *P* ≤ 0.01. Ranging from the rhizosphere to the endosphere, the number of OTUs in fungal communities showed a decreasing trend. The OTU numbers of different root compartments in continuous cropping were significantly higher than those in rotation.

**Fig 2:**
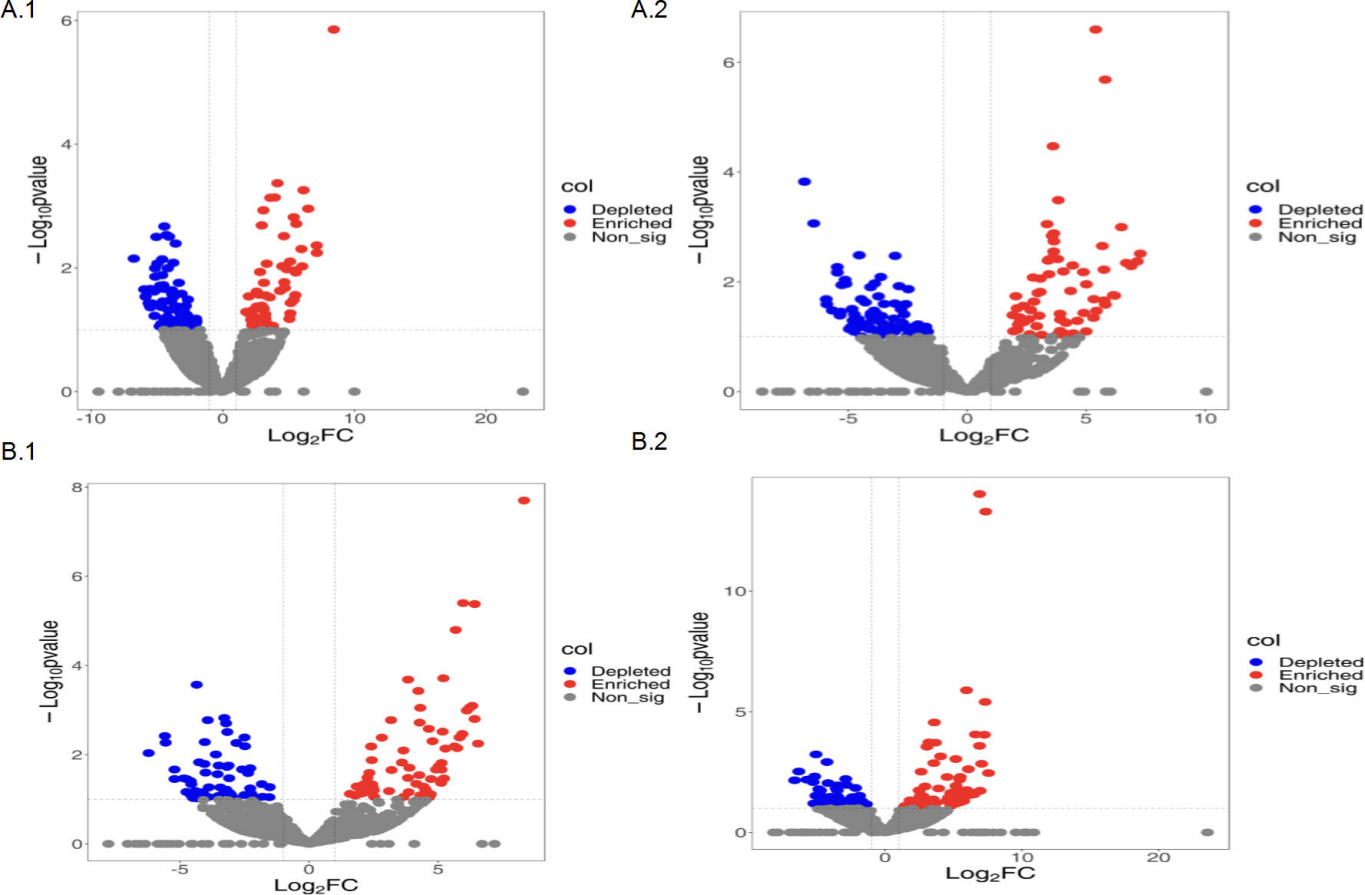
The number and enrichment/consumption of OTUs in three soybean root niches in continuous cropping soybean and corn-soybean rotation. (A) The accumulation and consumption of OTUs in the fungi in the rhizosphere, rhizoplane, and endosphere in the continuous cropping treatment. (A.1) Continuously cropped root surface. (A.2) In the continuously growing root. (B) In the rotation treatment, enrichment of OTUs and fungal consumption in the rhizosphere, rhizoplane, and endosphere. (B.1) In the continuously growing endosphere. (B.2) In the continuously cropped roots, red represents enriched OTUs, blue represents consumed OTUs, and gray represents unchanged OTUs (*P* < 0.01).

Different OTUs were seen in different root compartments. There were significant differences between enriched and depleted OTU species in different root compartments under the different treatments. Under continuous cropping, *Amanita*, *Mortierellales*, *Stephanosporaceae,* and *Mortierella* were the most abundant enriched fungal species, whereas *Lobulomycetales* and *Glomeraceae* were the most abundant fungal species that were depleted in the rhizoplane region. *Basidiobolus*, *Allomyces,* and *Mycoleptodiscus* were the most abundant enriched fungal species, whereas *Spizellomyces*, *Stropharia,* and *Lobulomycetales* were the most abundant depleted fungal species in the endosphere. In rotation, *Stropharia*, *Tremellodendropsidales,* and *Chaetomiaceae* were the most abundant, whereas *Ascomycota*, *Lentinellus,* and *Hypocreales* were depleted in the rhizoplane. The most abundant species were *Stropharia*, *Rhizophlyctis*, and *Sarocladium*, while *Lindtneria*, *Didymellaceae*, and *Tremellodendropsidales* were depleted in the endosphere(Fig. 2).

**Fig 3:**
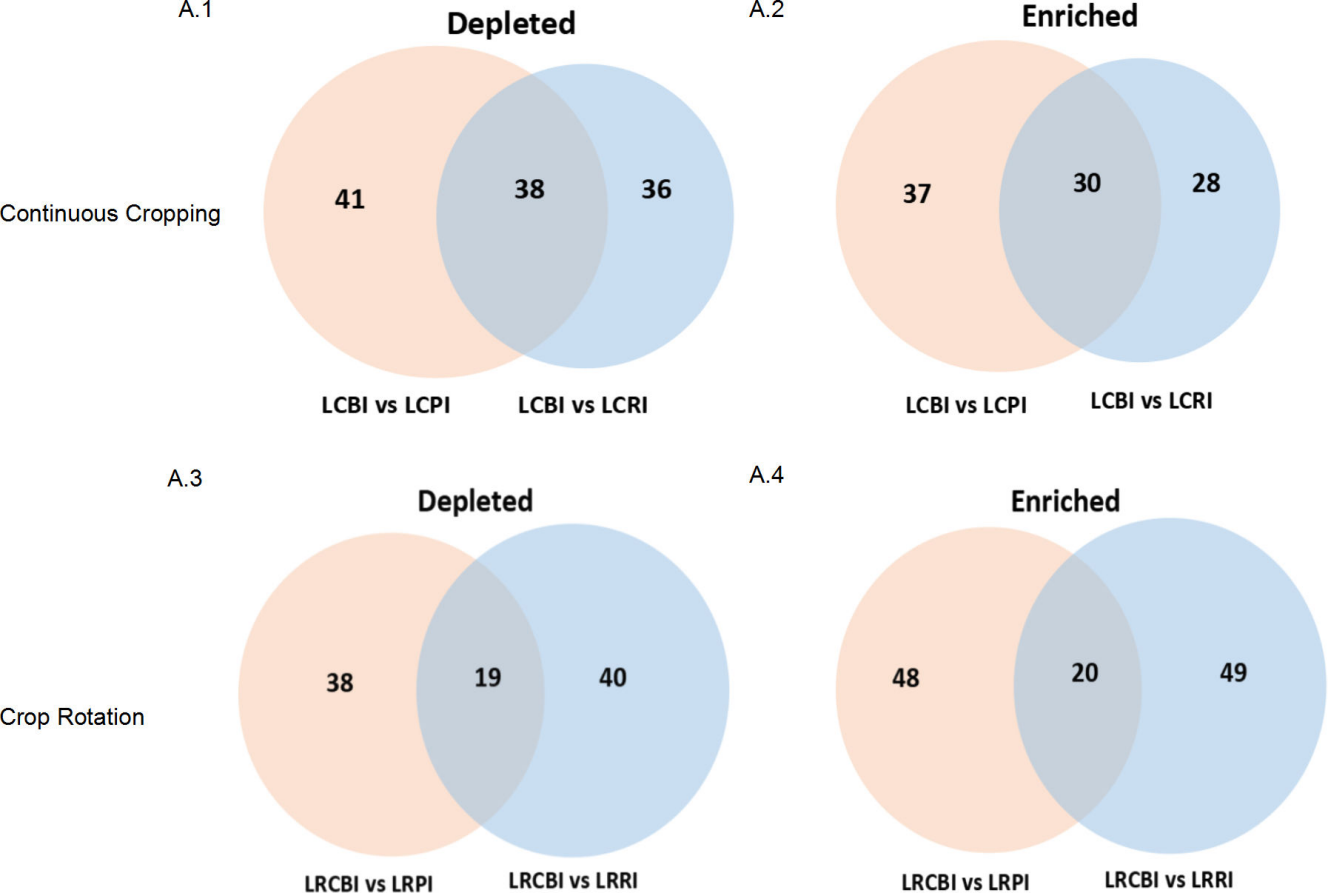
(A.1–A.2) Numbers of differentially enriched and depleted OTUs between each compartment compared with the rhizosphere of continuous cropping. (A.3–A.4) Numbers of differentially enriched and depleted OTUs between each compartment compared with the rhizosphere of crop rotation.

In the rhizoplane, enriched OTUs from continuous cropping were lower than those from rotation, but depleted OTUs showed an entirely different pattern (Fig. 3). In the endosphere, depleted OTUs from continuous cropping were higher than those from rotation, but there was no difference in enriched OTUs. Abundant fungi OTUs overlapped in different root compartments under different treatments: 51.7% and 44.8% of rhizosphere-enriched fungal OTUs were enriched in the rhizoplane and endosphere in continuous cropping, whereas 29% and 29.4% of rhizosphere-enriched fungal OTUs were enriched in the rhizoplane and endosphere in rotation. This suggested that microbes in the rhizosphere colonized the root.

### Co-occurrence network analysis

**Fig 4:**
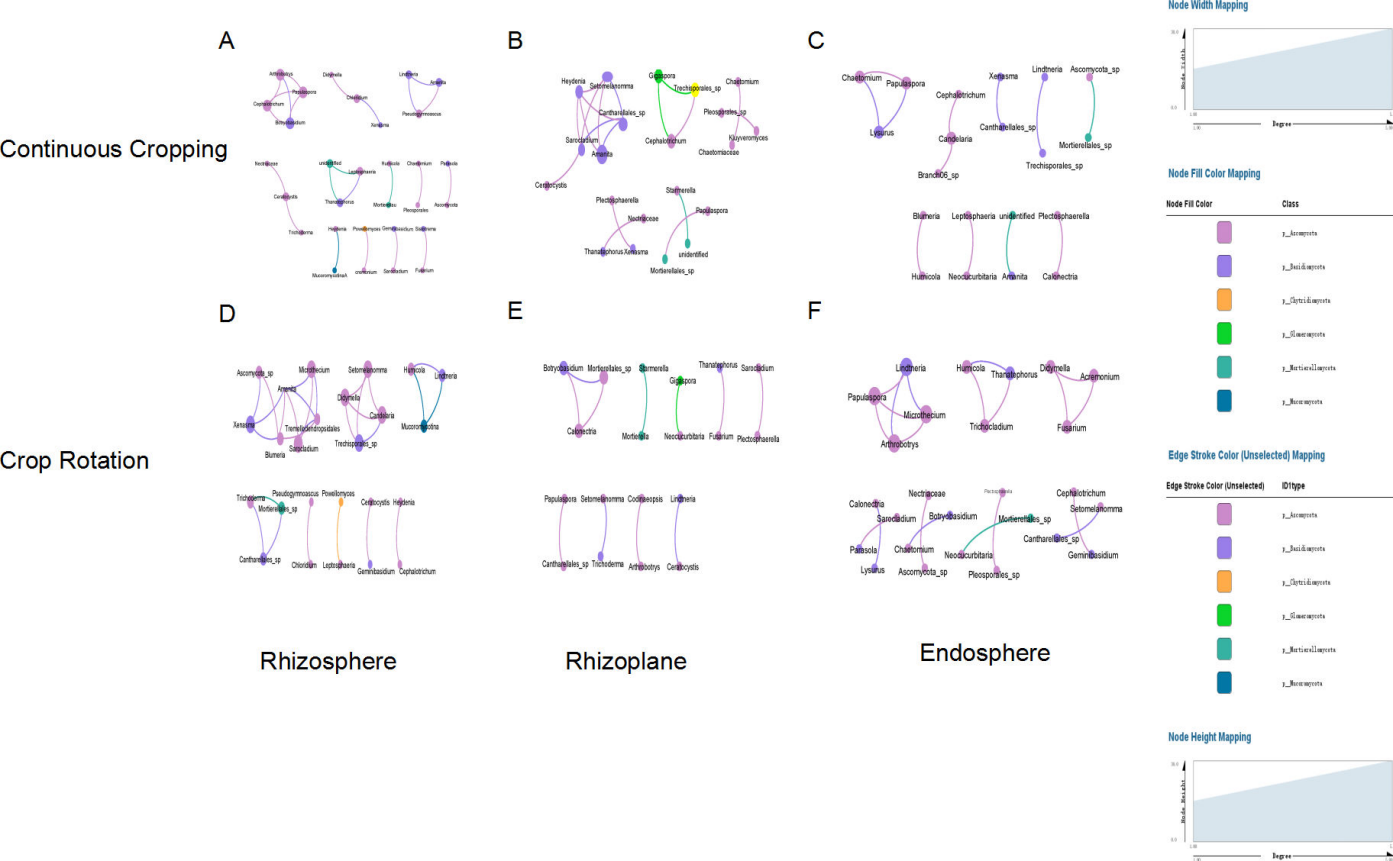
Fungus genus co-occurrence network of the following three root niches under continuous rotation and cropping. (A) Continuous cropping rhizosphere. (B) Continuous cropping rhizoplane. (C) Continuous cropping endosphere. (D) Rotation rhizosphere. (E) Rotation rhizoplane. (F) Rotation endosphere. Different nodes represent different genera, the node size represents the connectivity of the genus, the same color represents the same level of the door, and the connection thickness between nodes is positively related to the absolute value of the species interaction correlation coefficient (*P* < 0.05).

We constructed a co-occurrence network based on species-species associations (SSAs) (30) across different compartments of the root. There were no significant differences between rhizosphere and rhizoplane nodes and edges in continuous and rotational croppings. The number of endosphere nodes and edges under rotational cropping was significantly higher than that under continuous cropping, indicating that rotational cropping had more connections compared to continuous cropping. In the soybean rhizosphere soil treated with continuous cropping, *Fusarium* showed a negative correlation with *Stropharia*, while *Fusarium* showed a positive correlation with *Mortierella*. Rotation treatment eliminated the negative correlation between *Fusarium* and *Stropharia* in soybean rhizosphere soil, while *Fusarium* showed a negative correlation with *Mortierella, Calonectria,* and *Gigaspora*. Continuous cropping treatment resulted in a positive correlation between *Fusarium* and *Didymella* in the soybean root table, but this correlation did not exist in the soybean root table during rotation. Continuous cropping can cause changes in the correlation between pathogenic *Fusarium* and other pathogenic bacteria in soil (31), indicating that continuous cropping has to some extent increased the abundance of soil pathogenic bacteria. Most of the fungi under continuous cropping and rotation were *Basidiomycetes* and *Ascomycetes. Mortierella* fungal enrichment abundance in continuous cropping was significantly greater than that in the rotation, and the abundance of *Mucor* fungal enrichment in rotation was significantly higher than that in continuous cropping (Fig. 4).

### Functions of fungal communities associated with roots in different root compartments

**Fig 5:**
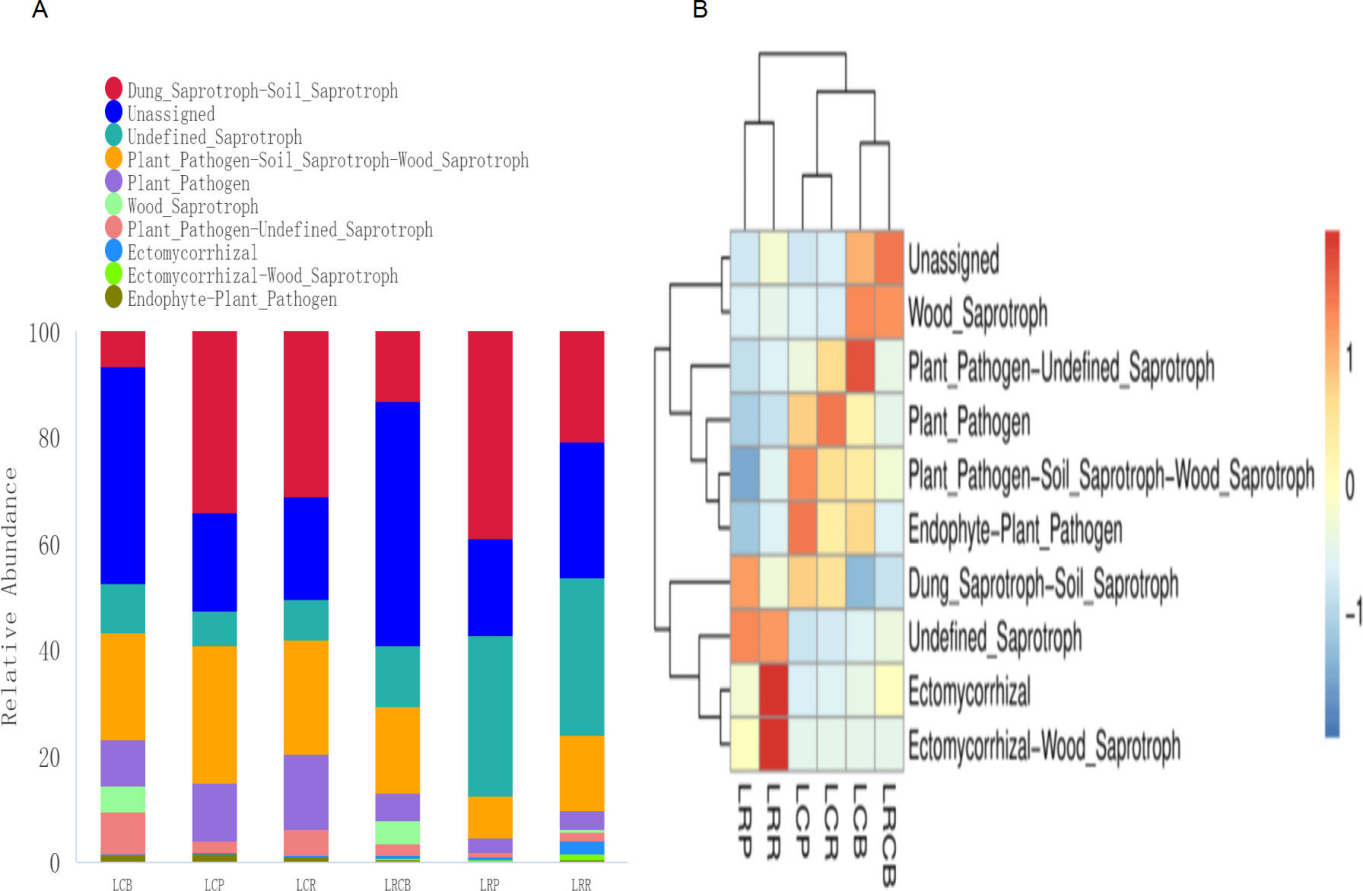
(A) Histogram of the top 10 trophic types of fungi in the genus level. (B) Heat maps of all predicted functional classifications of fungi in the top 10 genera (*P* < 0.05).

These changes in fungal communities further impacted fungal function. For this purpose, we analyzed fungal function in different root compartments on the basis of FUNGuild. Eight trophic modes were detected in this study, and the saprotroph is the most abundant, followed by pathotroph-saprotroph, pathotroph, symbiotroph, pathotroph-saprotroph-symbiotroph, pathotroph-symbiotroph, saprotroph-symbiotroph, and pathogen-saprotroph-symbiotroph (Fig. 5A). The relative abundance of saprotroph increased significantly in rotation cropping, while the relative abundance of pathotroph-saprotroph and pathotroph was lower than that in continuous cropping. There were also significant differences in the relative abundances of different fungal functional guilds among the different root compartments (Fig. 5B). The major foci of LRP were undefined_Saprotroph and Dung_Saprotroph-Soil_Saprotroph. Ectomycorrhizal-wood_saprotroph and Ectomycorrhizal were the main focus of LRR. LCP was mainly enriched in Endophyte-Plant_Pathogen and Plant_Pathogen Soil_Saprotroph-Wood_Saprotroph. LCP was mainly enriched in Endophyte-Plant_Pathogen and Plant_Pathogen Soil_Saprotroph-Wood_Saprotroph. LCR was primarily enriched in Plant_Pathogen. LCB was mainly enriched in Plant_Pathogen-Undefined_Saprotroph. LRCB was mainly enriched in Wood_Saprotroph and Unassigned. In addition, the relative abundance of potential pathogenic fungus was higher in the functional groups of LCB, LCP, and LCR, while the relative abundance of potential beneficial fungus was higher in the functional groups of LRP, LRR, and LRCB (Table 1).

**TABLE 1:** Taxonomy of all predicted known potentially beneficial and harmful fungi in the top 10 genus levels

Groups	Potentially beneficial fungus	Potentially harmful fungus
LCB		*Didymella*
LCR	*Thanatephorus*	*Calonectria, Plectosphaerella,* *Leptosphaeria, Powellomyces, Lectera*
LCP		*Cladosporium, Fusarium*
LRCB	*Lindtneria*	
LRR	*Sistotrema, Chloridium*	*Amanita*
LRP	*Stropharia, Geminibasidium,* *Arthrobotrys, Trichocladium,* *Mortierella, Cladophialophora, Lysurus*	*Codinaeopsis, Parasola*

### Differences in the metabolism of soybean soil in continuous and rotational croppings

**Fig 6:**
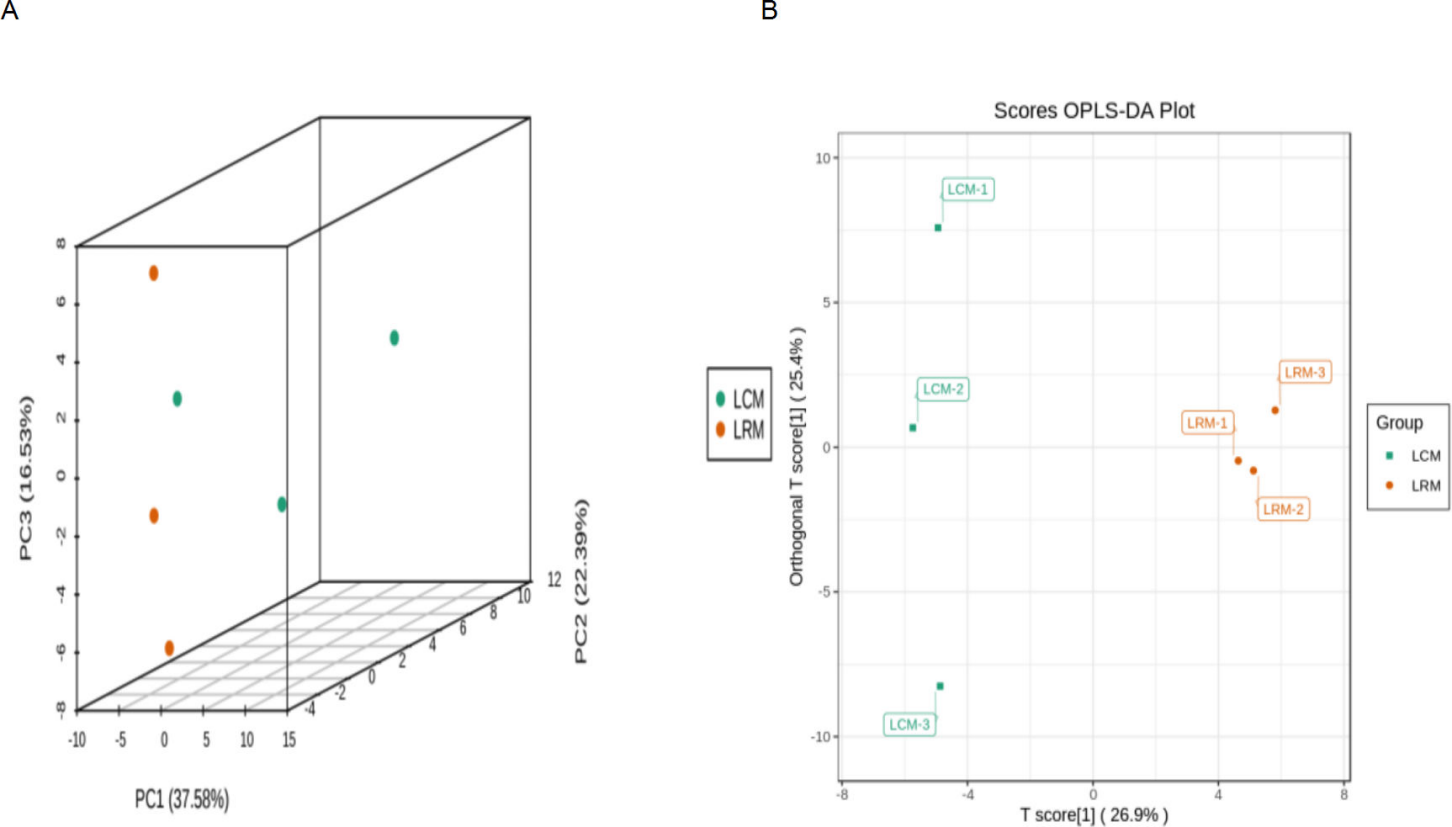
(A) PCA of the difference between two treated samples (LCM, continuous cropping soil differential metabolite; LRM, rotation soil differential metabolite) (*P* < 0.05). (B) OPLS-DA was used to analyze the difference between two treatment samples (LCM, continuous cropping soil differential metabolite; LRM, rotation soil differential metabolite) (*P* < 0.05).

The supervised discriminant analysis statistical method OPLS-DA model showed that rhizosphere soil metabolite composition was separated from one another between the different treatments, indicating that there were significant differences between continuous cropping and rotation. PC1, PC2, and PC3 dimensions accounted for 75.5% of the metabolite differences (Fig. 6), respectively. The top five metabolites were lipid (25.8%), acid (17.5%), alcohol (11.6%), carbohydrate (13.3%), and amine (7.5%).

To determine which metabolite changes derived the difference, we filtered out the different metabolites between continuous cropping and rotation. Compared with continuous cropping, rotation decreased the root metabolites glycerin, stigmasterol, eicosanoic acid, (Z)-9-octadecenamide, pentadecanoic acid, galacto heptose, sorbitol, and myo-inositol. The contents of 2,3-dihydroxypropyl dihydrogen phosphate (DDP), D-arabinol, 1,5-pentanediamine, and carbonic acid decreased, while sucrose, D-pinitol, benzyl alcohol, propanedioic acid, malonic acid, and hydroxyurea increased.

**Fig 7:**
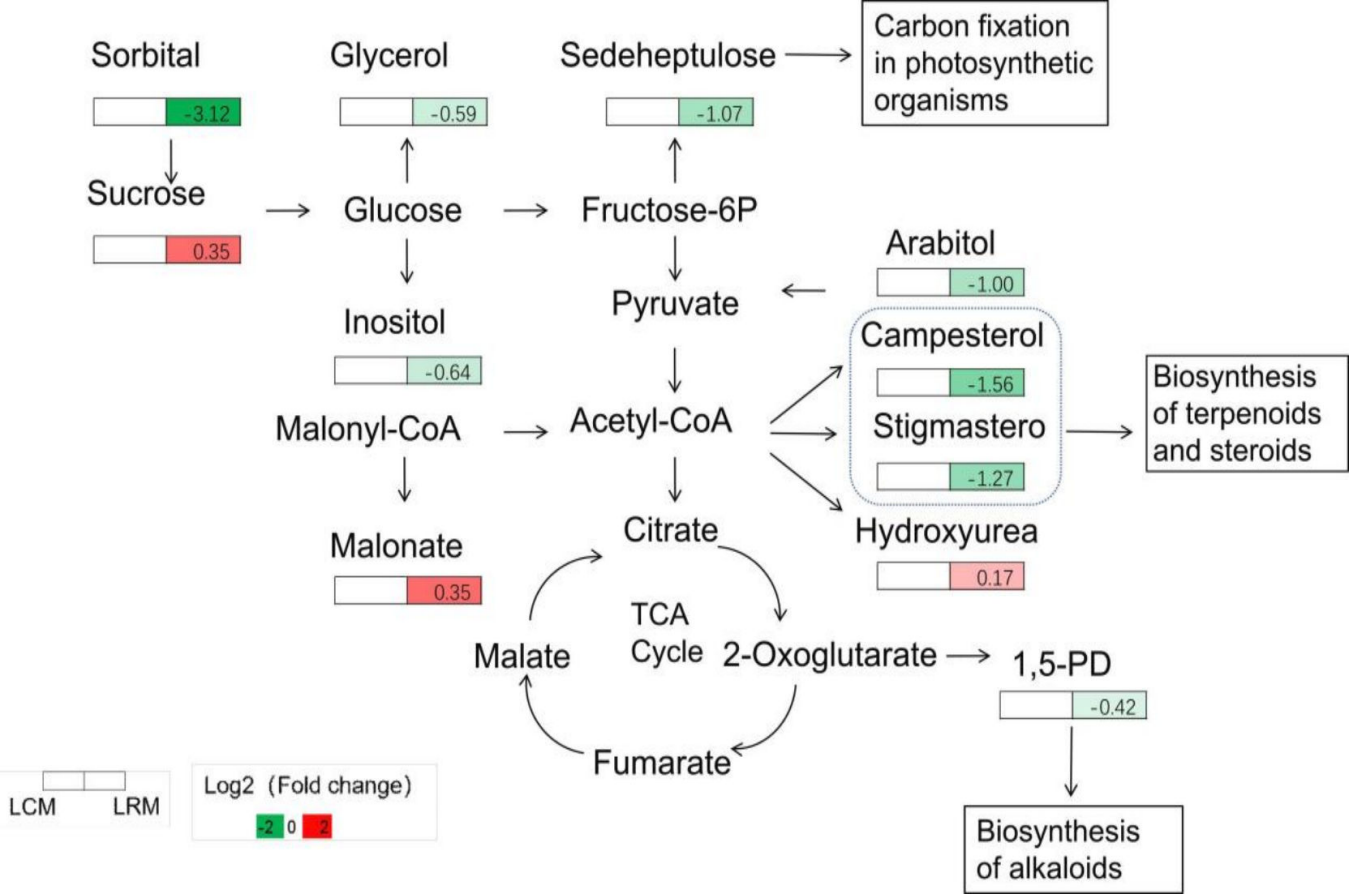
Differential metabolite metabolic pathway map (*P* < 0.05).

To further explore the effect of continuous cropping soybean on soil metabolites, we mapped a metabolic pathway comprising 11 differential metabolites by querying the KEGG database (Fig. 7). They were involved in the metabolism of carbohydrates, amino acids, fungi, photosynthetic carbon fixation, and other metabolic pathways. The sorbitol content of sorbitol under continuous cultivation was significantly higher than that in rotation, achieving a value 3.12 times that of the control. The content of sucrose and hydroxyurea in rotation was 0.35 and 0.17 times greater than that in continuous cropping.

### Correlations between soil metabolism and bacterial community in continuous and rotation croppings

**Fig 8:**
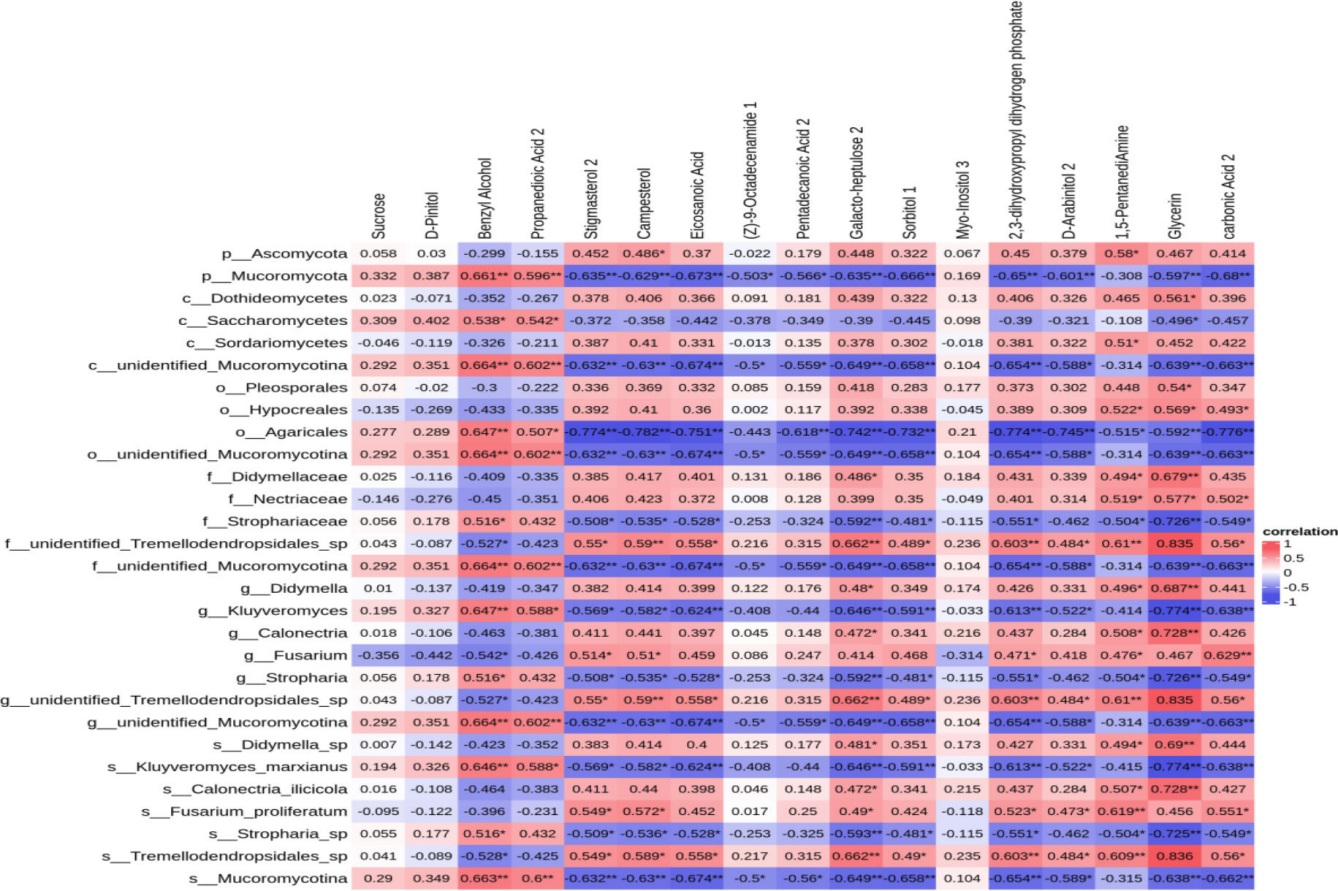
Correlating the relative abundance of genus-level differential fungi with differential metabolites. Vertical axes represent different fungal genera (based on ITS gene sequence). Horizontal axes represent differential metabolites. The red and blue bars denote the positive and negative correlations, respectively. * and ** represent significant (*P* < 0.05) and extremely significant (*P* < 0.01) effects, respectively.

Selection of differential fungi based on changes in fungal abundance during the fungal community diversity process and correlation analysis with differential metabolites in the soil. Twenty-nine different fungal species were found to be associated with different metabolites. There was a significant correlation between the fungus *Dothideomycotes* and the glycerin metabolite (c = 0.561, *P* = 0.015). There was a significant correlation between *Saccharomycetes* and both propanedioic acid 2 (c = 0.542, *P* = 0.020) and glycerin (c = −0.496, *P* = 0.036). A significant correlation was found between *Sortariomycetes* and 1, 5-pentanediamine (c = 0.510, *P* = 0.031). *Mucor mycotina* was found to be significantly correlated with benzyl alcohol (c = 0.664, *P* = 0.003) and eicosanoic acid (c = −0.674, *P* = 0.002). *Didymellaceae* was found to have a significant correlation with glycerin (c = 0.679, *P* = 0.002). *Neuroceae* was significantly correlated with glycerin (c = 0.577, *P* = 0.012). *Strophariaceae* was significantly correlated with benzyl alcohol (c = 0.516, *P* = 0.028) and glycerin (c = −0.72, *P* = 0.0007). There was a significant correlation between *Fusarium* and both carbon 2 acid (c = 0.629, *P* = 0.005) and 1, 5-pentadiamine (c = 0.619, *P* = 0.006). The correlation between *Calonectria* and glycerin was significant (c = 0.728, *P* = 0.0006). Based on the above research findings, it was possible that these fungi were involved in the formation of the aforementioned metabolites (Fig. 8).

## DISCUSSION

Although many factors were related to barriers to continuous cropping soybean, the soil sterilization experiment showed that it improved soybean growth in continuous cropping. Liu (10) reported that biotic, rather than abiotic, factors were the primary reasons for the continuous barriers to soybean. Soil microbes are important in promoting the decomposition of organic matter, nutrient cycling, plant heathland, and growth promotion (32), which are useful for soil health (33). Some studies reported that continuous cropping altered the structure of the fungal community and caused pathogen emergence, which was known to inhibit the growth of plants (34, 35). Soil rich in fungal pathogens not only affects the growth of plants and decreases plant biomass but also decreases the production of soil-cultivated mushroom crops such as morels (36). The process of fungal community diversity along the soil-root continuum, however, remained largely unknown. A better understanding of the fungal community diversity process of the root microbiome provided more critical insights into plant, soil, and microbial interactions (37).

### Effect of continuous cropping on fungal microbial assemblage

Plant roots recruited specific microorganisms to the rhizosphere from the bulk soil, and microorganisms formed complex co-associations with plants and have been shown to have important roles in promoting plant productivity and health in natural settings (17). Plant species and genotypes played an important role in this process alongside environmental and edaphic drivers (16, 19, 38); we partitioned the environment comprising soil and plant root into three compartments in order to observe the process of endosphere microbiome acquisition; we found that the bacterial and fungal community diversity of the different compartments was different; and rhizoplane acted as a selective gate controlling the entry of microbes into the root. This study showed that fungal communities in different root compartments under both continuous and rotational cropping were different; there was a gradual decline in fungal diversity from the rhizosphere to the endosphere, and enrichment or depletion of fungal OTUs across rhizocompartments indicated that colonization of soybean root fungi was not a passive process; plants had the capacity to select certain microbes from different root compartments. At the phyla level, the abundance of *Basidiomycota* increased gradually from the rhizosphere to the endosphere both under soybean continuous cropping and maize-soybean rotation, whereas the abundance of *Ascomycota* decreased. The enrichment of certain fungi (such as *Mucoromycota* and *Chytridiomycota*) in the LRCB was highest. At the genus level, the enrichment of *Fusarium* and *Lysurus* in the endosphere was greater than that in the other compartments, but the enrichment of *Stropharia* in the LRR and LRP was greater than that in the others. All of these results suggested that the fungus was attracted to a suitable niche (35), and continuous cropping altered the fungal assemblage. The enriched OTUs from continuous cropping were lower than those from rotations, but the depleted OTUs from continuous cropping were higher than those from rotations. A higher proportion of intercropped rhizosphere fungi were colonized at the root compared to the crop rotation, indicating that continuous cropping had a greater influence on root fungal colonization than crop rotation. Some noxious fungi (such as *Fusarium*, *Calonectria*, *Didymella*, *Setomelanomma*, *Parasola*, *Cladosporium*, *Histoplasma*, *Leptosphaeria*, *Lectera,* and *Basidiomycota*) were found in greater relative abundance in three root compartments in continuous cropping than those in rotation. Some noxious fungi (such as *Plectosphaerella* and *Codinaeopsis*) in the rhizosphere in continuous cropping were higher than those in rotation but did not differ in other root compartments. All of these findings suggested that continuous cropping increased soil pathogens, but only partial soil pathogens were able to colonize from the rhizosphere to the endosphere. These soil pathogens were able to grow rapidly on their host and infect the host root leading to a decline in plant biomass (35, 39). In comparison to rotation, continuous cropping decreased the relative abundance of certain beneficial fungi (such as *Stropharia*, *Kluyveromyces*, *Mucoromycotina*, *Arthrobotrys*, *Trichocladium*, *Thanatephorus*, *Chloridium*, *Oidiodendron*, *Nectria*, *Sonoraphlyctis*, *Nectriaceae,* and *Coniochaeta*) in three root compartments, which were found to be related to disease resistance, nutrient utilization, and so on.

### Effect of continuous cropping on microbial metabolism

Soil metabolites were derived primarily from plant roots, and microorganisms were carried out by plant species, genotype, fertilizer, and crop (40, 41). Changes in soil metabolite composition and content revealed the direct or antecedent response of soil microorganisms to the soil environment (3). Cultivation resulted in changes not only to the structure of the soil microbial community but also in the metabolites (42 – 44). In our study, continuous cropping significantly affected the spectrum of soil metabolites, including lipid, acid, alcohol, carbohydrate, and amine, and subsequently interfered with certain metabolic pathways, including sugar, amino acid metabolism, fungal metabolism, the fixation of photosynthetic biological carbon, and the like.

In the rhizosphere, the increase in organic acids, sugars, and amino acids enhanced the growth of the pathogens. Organic acid metabolism in the soil was a major cause of soil self-toxicity (45). The results of this study showed that the content of eicosanoid acid and pentadecanoic acid in continuous cropping was higher than that in rotation, and both eicosanoid acid and pentadecanoic acid were found to be antifungal organic acids that were positively correlated with the number of fungal pathogens in the soil (46). Sugars provided a potential source of carbon for microbes in the rhizosphere (47). Continuous cropping brought about changes in the sugar and organic acid content of plant root exudates (48). Galacto-heptulose and sorbitol were polyols, and the enriched polyols didn’t affect the normal growth of microorganisms and cells and also increased the ability of plants to withstand stress. Soybeans coped with a variety of stresses through polyol accumulation. On the contrary, the high metabolic content of sorbitol caused an abnormal fungal community of plant roots and induced necrosis of the plant (49). Phytosterols were important in plant-environment interaction, and previous studies showed that the content of myristerol in cotton roots was significantly increased following inoculation with plant pathogens (50). This study showed that the contents of myristerol, campestral, canosterol, and sorbitol in continuous cropping were higher than those in rotation, which could be an important reason for the development of continuous soybean disorders. Benzyl alcohol is an antimicrobial compound, and it affects the cell membrane of the fungus, leading to fluidization or perturbation of membrane protein function (51). In this study, the benzyl alcohol content in rotation was higher than that in continuous cropping, which indicated that the changes in the fungal cell membrane caused by benzyl alcohol enabled other antibacterial compounds to penetrate the cell membrane with ease. In this study, myo-inositol content in continuous cropping was higher than that in rotation. Myo-inositol was an important mediator in plant-soil interactions (44).

There was a significant correlation between metabolite composition and microbial communities in soil (41, 52). Soil microbiota were important executors of soil metabolic activities, and this was a direct reflection of the detectable biological responses of soil microorganisms in a variety of conditions (53). Here, we chose differential fungi on the basis of changes in fungal abundance during the assembly process, and then, the correlations between differential fungus and differential soil metabolites were analyzed. We found that the process of mushroom fungal community diversity was significantly correlated with soil metabolites. For example, soil pathogen fungal community diversity was closely linked to sugar and acid metabolism.

### Conclusion

In this study, microbiology with non-target metabolomics was combined to explore the ecological diversity process of the fungal community of different root compartments (rhizosphere, rhizoplane, and endosphere) between soybean continuous cropping and maize-soybean rotation. Continuous cropping significantly increased fungal community diversities in different root compartments and changed their composition, enrichment, and depleting processes. However, only partial soil pathogens colonized from the rhizosphere to the endosphere, which suggested that root compartments had selective effects on root-associated fungal community diversity. Moreover, metabolomics analysis showed that soybean continuous cropping significantly changed the soil metabolite spectrum, and the differential metabolites were significantly enriched to carbohydrate, amino acid, fungal metabolism, photosynthetic carbon fixation, and other metabolic pathways. Correlation analysis displaying soil metabolite composition was a main factor that affected fungal community diversity.

## Data Availability

The metabarcoding data sets of fungal ITS amplicons are accessible under NCBI BioProject PRJNA1001153.
